# Can Patient Navigators Help Potential TB Patients Navigate the Diagnostic and Treatment Pathways? An Implementation Research from India

**DOI:** 10.3390/tropicalmed6040200

**Published:** 2021-11-15

**Authors:** Tushar Garg, Vikas Panibatla, Joseph P. Carel, Achanta Shanta, Manish Bhardwaj, Miranda Brouwer

**Affiliations:** 1 Innovators In Health, Patna 800001, India; mbhardwaj@innovatorsinhealth.org; 2 TB Alert India, Hyderabad 500001, India; vikass@tbalertindia.org; 3 Independent Consultant, New Delhi 110001, India; carel.joseph@gmail.com (J.P.C.); shanta010@gmail.com (A.S.); 4 PHTB Consult, 5018 Tilburg, The Netherlands; mbrouwer@phtbconsult.org

**Keywords:** patient navigation, active case finding, community health worker, accredited social health activist, person centered care

## Abstract

Navigating the Indian health system is a challenge for people with tuberculosis (TB) symptoms. The onus of organizing care is on the patient and their families alone. Factors like gender discrimination and opportunity costs further aggravate this. As a result, people may not complete the diagnostic and treatment pathway even though they experience poor health. Navigators can aid in the pathway’s completion. We implemented two projects in India—a public sector intervention in Bihar, with a population of 1.02 million, and a private sector intervention in Andhra Pradesh (AP), with a population of 8.45 million. Accredited Social Health Activists (ASHAs) of the public health system in Bihar and local field officers in AP facilitated the patients’ navigation through the health system. In Bihar, ASHAs accompanied community-identified presumptive TB patients to the nearest primary health center, assisted them through the diagnostic process, and supported the patients throughout the TB treatment. In AP, the field officers liaised with the private physicians, accompanied presumptive patients through the diagnosis, counseled and started treatment, and followed-up with the patients during the treatment. Both projects recorded case-based data for all of the patients, and used the yield and historical TB notifications to evaluate the intervention’s effect. Between July 2017 and December 2018, Bihar confirmed 1650 patients, which represented an increase of 94% in public notifications compared to the baseline. About 97% of them started treatment. During the same period in AP, private notifications increased by 147% compared to the baseline, and all 5765 patients started treatment. Patient navigators support the patients in the diagnostic and treatment pathways, and improve their health system experience. This novel approach of involving navigators in TB projects can improve the completion of the care cascade and reduce the loss to follow-up at various stages.

## 1. Introduction

Globally, the TB care cascade has shown promising improvements in the past years. The reduction in missing notifications and the proportion of undiagnosed cases is improving the case-finding [1]. However, the loss to follow-up at various stages of TB care prevents expeditious progress to eliminate TB [2]. In addition, the quality of TB care is not adequate everywhere, which limits people from accessing adequate TB services. People-centered care in health systems can address some of these issues [3].

The public and private health systems in India have a fragmented structure from both the care-seeking and care-delivery perspectives [4]. Navigating through their complex processes with multiple steps spread amongst dispersed institutions is an onerous exercise [5]. Even when patients access such services under one common roof at a health center, it is their responsibility to organize the care. In the long duration of TB care, patients face these challenges during both diagnosis and treatment. Diagnosis requires visiting multiple sites for different tests, and repeated visits to the diagnostic centers and the consulting physician [6,7]. The treatment phase involves repeated visits for directly observed treatment if it is not organized in the community, as well as several medical monitoring visits [8]. Furthermore, patients face problems outside the health system. Patients brave social challenges like stigma, gender discrimination and patriarchy; structural barriers like the unavailability of transport; and financial impediments like opportunity costs and the steep financial costs of healthcare [9,10,11,12,13,14,15]. 

Patient navigators hold a promise for solving some of these challenges, and for eventually improving a patient’s experience of TB care [16,17]. They can lower social, economic, knowledge, and health system barriers [18,19]. Experience from specialties with complex and prolonged treatment suggests their utility in improving patient care [20,21]. In the Indian context, community health workers (CHW) can serve as patient navigators [22]. We implemented two TB projects in India with CHWs as patient navigators, closing the gap between the health system and communities, which is an innovative approach in our settings. 

The aim of this paper is to describe our experience with CHWs as patient navigators to improve TB care in both public and private sector TB projects in India.

## 2. Methods

We implemented two TB projects in partnership with the National TB Elimination Program (NTEP) for patients seeking care in the public and the private sector in two different states of India. Both projects had various interventions across the TB care cascade. Community health workers (CHW) formed the backbone of the interventions, and supported the organization of the patient care. 

### 2.1. Study Design

We conducted a retrospective analysis to study the effects of the intervention over 15 months between July 2017 and December 2018. Our study built on the TB REACH framework to evaluate case-finding implementations [23].

### 2.2. Project Details

#### 2.2.1. Public Sector project

##### Study Setting

We implemented the project in three blocks of the Samastipur district in Bihar, India. Together, the Bibhutipur, Ujiarpur and Sarairanjan blocks have a combined population of 1,021,483. Nearly 87% of the population is rural, and agriculture is the chief occupation of 55% of the population [24]. The literacy rate is 64%, and the female literacy rate is lower by 20%. The highest earning member in 70% of the households earns less than INR5000 [25].

The infant mortality rate and maternal mortality ratios are 53 per 1000 live births and 280 per 100,000 live births, respectively [26]. Only 10% of the pregnant women in the district undergo all four antenatal checkups, and 73% deliver in a health facility [27].

Each block has a health center, which serves as a Basic Management Unit (BMU) in the NTEP, with four operational designated microscopy centers (DMC). A Senior Treatment Supervisor (STS) manages each BMU, and laboratory technicians run the DMCs. The public sector case notification and pre-treatment loss to follow-up in the district in 2017 was 55 per 100,000 population and 25%, respectively [28]. The treatment success rate for patients notified in 2017 in the public sector in the district was 75% [29].

##### Intervention

The key intervention comprised community-based active case finding and treatment support using CHWs called Accredited Social Health Activists (ASHA). The ASHAs were supported by field coordinators (FC), with each FC covering a catchment of 50,000 of the population, serviced by 35—35 ASHAs. A block coordinator supported 6–7 FCs each. A team of project managers supervised the overall project. The ASHAs received an activity-based incentive, whereas the project staff received a fixed salary comparable to the corresponding position in the public health system. Each level was supervised and supported through regular weekly and monthly meetings that focused on routine reporting and training, and project review, respectively. The project staff interfaced with the corresponding officials in the national TB program regularly. The organizational details have been described previously [30].

The trained ASHAs identified and listed people eligible for screening (called referrals) while performing their routine work in the community, of which they have a comprehensive understanding of the health status. The project’s field coordinator then screened the people referred for the following TB symptoms: a cough of ≥2 weeks or a productive cough, any instance of hemoptysis in the last 6 months, any instance of chest pain in the last 1 month, a fever of ≥2 weeks, night sweats for ≥2 weeks, severe weight loss in the last 3 months, and swelling in a lymph node. A presumptive TB person had at least one symptom on the screening. 

The ASHA accompanied all of the presumptive people to the health center for a physician consultation, sputum microscopy and a chest X-ray (CXR), for which the presumptive people received a transport allowance. The ASHA also screened any person referred by the rural medical practitioners (RMP, an informal provider). If the microscopy was positive or the CXR was abnormal, we used a GeneXpert test. Furthermore, if both the microscopy and GeneXpert were normal, the physician could also order a GeneXpert based on the clinical presentation. For extra-pulmonary TB, the physician could request additional diagnostics such as ultrasonography, fine-needle aspiration and cytology, or a bone X-ray. Based on the results of the diagnostic tests, the physician diagnosed TB, notified the patient, and, subsequently, initiated treatment. 

The ASHA received the drugs from the STS and undertook the initial home visit for the treatment initiation and counseling with the patient and the family at their home. Consecutively, the ASHA delivered the drugs to the patients each month, monitored for adverse effects, ensured adherence and the follow-up examination, and maintained the records. All of the diagnostics and treatment were free of cost to the patient, and the ASHAs supported the patients at each step in this care pathway as an advocate, advisor, and counselor. Within the project, for each confirmed case of TB, the ASHA received INR200 (USD3; USD1 = INR67) for the referral and INR300 (USD5) for supporting their diagnosis. Furthermore, they received INR1000 (USD15) for supporting the treatment of each TB patient.

#### 2.2.2. Private Sector Project

##### Study Setting

We implemented the project in two BMUs each in the Chittoor and Kurnool districts of Andhra Pradesh, India. The combined population of the study area was 4,242,283, with a 20% urban population [24]. The literacy rate was 77%. The infant mortality rate per 1000 live births and the maternal mortality ratio per 100,000 live births in the region were 29 and 74, respectively [31,32].

The NTEP had a similar structure, as explained above. The private case notification rate per 100,000 of the population in the study region was 43 in 2017 [28]. The treatment success rate was 95% in the private sector for patients diagnosed in 2017 [29]. 

##### Intervention

The purpose was to increase the case-finding in the private sector by screening more patients for TB, improving diagnosis, and notifying the TB patients. The project established five diagnostic hubs in the existing private diagnostic centers which were preferred by the private providers and equipped with florescent microscopy and GeneXpert. In addition, Community Health Workers (CHW) liaised with the private providers to ensure data collection and referral to the diagnostic hub. For every 25–50 private providers, one CHW handled the follow-up and coordination. The CHWs were paid INR100 (USD1.5) as an incentive per referred presumptive person if that person presented at the diagnostic hub. For a subsequent TB diagnosis and notification by the provider, the incentive for the CHW was INR200 (USD3). All of the CHWs in a district were monitored by District Coordinators, who were paid staff on a par with the Senior Treatment supervisors (STS) in the NTEP. One Project Manager coordinated with 2 district coordinators. Supportive supervision was provided through regular field visits and fortnightly and monthly reviews.

The project mapped the private providers within the area of each diagnostic hub, and established a partnership. The providers identified people with presumptive TB based on symptoms, and advised them to be tested at their designated hub. The CHW at the clinic entered the referral information, issued a referral slip, and guided the people to the hub. In case the CHW was at a different health facility, the provider’s assistant contacted the CHW for the completion of this process. The people with presumptive TB provided a sputum sample at the diagnostic hub for smear microscopy. The patients also had a CXR at the diagnostic hub or at the providers’ radiology facility of choice.

The project established a socio-business model for GeneXpert testing in which private providers could order one free GeneXpert test for every 2–3 paid GeneXpert tests, each costing INR1800 (USD27). The CHW informed the provider of the test results and facilitated a patient with a confirmed TB diagnosis to visit the provider for treatment initiation if they received treatment from the private provider. The CHW linked the patients to NTEP for initiation if they preferred to receive the treatment in the public sector. The CHW notified the case to NTEP. The CHW followed up with the patients through phone calls and monitored the adherence. In addition, the project also incentivized the providers to notify the patients diagnosed with TB who did not have their diagnosis established through the diagnostic hub, with INR75 (USD1) and INR100 (USD1.5) for each clinically diagnosed and microbiologically confirmed case, respectively. The CHWs also received performance-linked remuneration.

### 2.3. Data Collection

TB REACH projects report baseline and quarterly data through an online platform, which includes both the project and NTEP data. The project data, called process indicators, follows the pathway of care as described by Blok et al [23]. We extracted the process indicators of the project, the NTEP notification data, and narratives from the quarterly reports of both of the projects.

We collected data on number of referrals, people screened, people identified with TB symptoms or presumptive TB cases, presumptive TB cases tested for TB, people identified with TB, people with microbiologically confirmed TB (Bac+), TB patients who started on treatment, and TB patients successfully treated. We accessed disaggregated notification data collected by the NTEP. We also extracted project narratives, which included project progress, a description of the activities, the interpretation of the quarterly data, and challenges in implementation.

### 2.4. Analysis

We used Microsoft Excel 2016® for the data analysis. The process indicators were used to build the pathway of care. Furthermore, we derived the pre-diagnostic loss to follow-up (PDLFU), the proportion of Bac+ cases, and the pre-treatment loss to follow-up (PTLFU). The PDLFU was the proportion of presumptive TB cases not tested for TB, and PTLFU was the proportion of people identified with TB who did not initiate TB treatment. We compared the NTEP notification rates with the historical rates. We used the project narrative to identify the implementation challenges and the solutions proposed. We analyzed the subsequent reports to assess whether the proposed solutions resolved the challenge. 

## 3. Result

### 3.1. Public Sector Project

**Figure 1:**
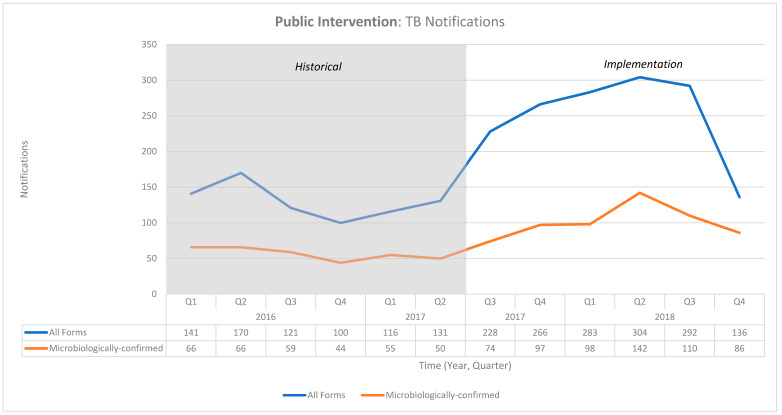
Graph of the notifications in the National TB Elimination Program in the public project intervention area between January 2016 and December 2018.

Between July 2017 and December 2018, the project reported 16,094 screenings. The screenings resulted in 14,832 presumptive TB cases, and 58% of those were tested for TB. A total of 1650 cases were diagnosed with TB, out of which 54% were microbiologically confirmed diagnoses. About 97% of the confirmed patients initiated TB treatment, and 95% completed treatment successfully. The pre-diagnostic loss to follow-up (PDLFU) and pre-treatment loss to follow-up (PTLFU) were 42% and 3%, respectively (Table 1). These activities resulted in an increase in all-form and microbiologically confirmed TB notifications of 94% and 79% in the public sector, respectively (Table 2) (Figure 1).

### 3.2. Private Sector Project

**Figure 2:**
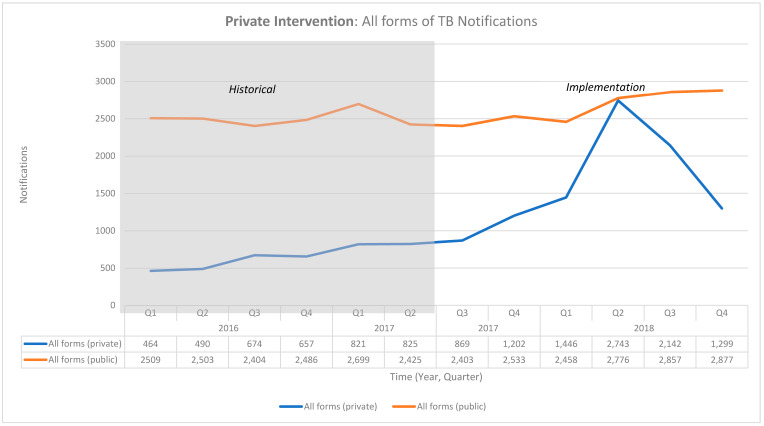
Graph of the notifications in the National TB Elimination Program in the private project intervention area between January 2016 and December 2018.

The project reported 12,041 presumptive TB cases between July 2017 and December 2018, which were all tested for TB. A total of 2403 cases were diagnosed with TB, out of which 91% were microbiologically confirmed cases. An additional 3362 patients were notified to the NTEP through the notification incentivization (Table 3). These activities resulted in an increase in all-form TB notifications of 6% in the public sector and 147% in the private sector (Table 4) (Figure 2). As shown in Figure 2, the numbers were reduced in the last quarter of the project due to reduced project activities. 

## 4. Discussion

Our results show that CHWs acting as patient navigators improved TB care in both public and private sector TB projects in India. The notifications increased substantially in both projects: 94% for all-form TB in the public project and 147% in the private project. Furthermore, the treatment initiation rate was high in both projects: 97% for all-form TB in the public and 100% in the private project. The corresponding initiation rates for patients in 2018 in the routine project were 93% and 100%, respectively [29]. While the two projects sustained the high treatment initiation rates with substantially higher notifications, the role of CHWs in the overall quality of TB care is also valuable.

A person-centered TB care paradigm entails actively acknowledging and addressing the needs of people who require TB care. While a hospital-based model of care recognizes the needs within a clinical setting, it is oblivious to the challenges faced outside of the hospital, which can adversely affect the access to and quality of TB care. In this context, the novel patient navigation approach showed in this paper aimed to solve the social and structural problems restricting quality TB care. Our innovation illustrates the feasibility of a CHW-led patient navigation program with promising results, as well as its applicability in high-burden, low-resource settings. The projects illustrate the utility of connecting various stakeholders in the TB care continuum. CHW’s connection to people with presumptive TB helps in accessing care and improves their experience. Similarly, connecting private providers to testing facilities improves the quality of care and patient experience.

Previous studies have also highlighted the potential of CHWs in improving TB care through improved case detection, improved linkage to care and improved treatment support [22]. In these studies, the CHWs in fact act as patient navigators to a large extent, as the CHWs in our projects did. In the public project, ASHAs encouraged the presumptive TB patient and their family to undergo the diagnostics by assuring them of their support, and addressed the stigma associated with TB and its testing. She accompanied the presumptive patients through the diagnostic process by assisting them in out-patient registration and being seen by the physician. After the TB diagnosis, she counseled the patient and the family, and started the treatment at their home itself. During the treatment, they made regular home visits to deliver drugs and ask after the patient’s health. They also monitored the adherence and adverse effects on such visits. As in the diagnosis stage, the ASHAs ensured adverse effect management and follow-up examination at the health facility.

In the private sector project, the CHWs followed the patient through the diagnostic process and supported the private providers administratively. They counseled the people with presumptive TB to undergo testing for TB. They also accompanied patients to the designated diagnostic hub for testing and facilitated in the sputum sample collection. After a TB diagnosis and subsequent treatment initiation, the CHW counseled the patient by phone on the importance of adherence to the prescribed treatment. Finally, they followed-up with the patient on monthly calls to monitor adherence and enquire after their health.

Patient navigators have proven their success in increasing participation in screening programs and subsequent follow-up care events [16]. It is in fact not surprising that a similar approach in TB care is also successful, given the challenges patients face [5].

The patient navigation role worked insufficiently in the public project in ensuring that all people with TB symptoms were tested for TB, which occurred in only 58% of those with symptoms (Table 1). Apart from the people who considered that their symptoms had improved after the screening, and hence testing was not necessary, a study by the project found that people considered misinformation and stigma, deficient family and health provider support, transport challenges and poor services in the public health system as major challenges to go through the diagnostic process [14]. This shows that some people need more than a supportive patient navigator to navigate the health system. 

The lessons learned from our projects were the need for the continuous training and supervision of the CHW, the recognition of the CHWs’ contribution in the form of incentives, and a continued interaction with the providers both in the public and private sectors. Our projects demonstrate a pathway for integrating patient navigation into the national TB program using existing CHW in the health system. Utilizing the existing human resource enhances the feasibility of a wide-scale implementation with minimal overheads. In fact, ASHAs—as CHWs—have a pivotal role in assisting patients in accessing TB services and improving their experience in the public project. Their contribution is corroborated by the drop in notifications in quarter four of 2018, resulting from a state-wide strike of ASHAs (Figure 1). Furthermore, our previous research from the same project has shown that involving CHWs like ASHAs in the TB project doesn’t affect their performance in other duties [30].

Patient navigation programs can be human resource intensive, thereby raising concerns around their sustainability considering the already-stretched budgets of TB programs in low-resource settings. There are two considerations in this aspect. First, the cost-effectiveness estimates from such programs will inform of their overall utility. Considering the societal perspective, these programs avert future costs by diagnosing and treating people with active TB whose health may worsen in the absence of prompt treatment, in addition to preventing new TB cases arising out of continued transmission. Second, national programs can consider leveraging already-existing human resources within the broader public health system, like ASHAs, thereby reducing the demand for additional resources. 

A strength of our study is that it occurred in the actual world and has a good potential to reflect reality. At the same time, this is a limitation, as other factors may have contributed to the results. However, given the wealth of evidence of the role of patient navigators, we consider this limitation a minor one.

In summary, we conclude that patient navigation is an innovative approach to improve TB program performance, and we encourage the national TB program to consider involving people in this role, whether that is an existing cadre such as the ASHAs or dedicated people with an adequate incentive. Important conditions—such as the training, supervision and continued engagement of providers, and sufficient funding—are crucial for successful implementation. In India, ensuring that people who start the TB care process finish this process can contribute significantly to the identification of the missing TB patients.

## Figures and Tables

**Table 1:** Outcome in the public project between July 2017 and December 2018.

Indicators	2017		2018				Total
	Q3	Q4	Q1	Q2	Q3	Q4	
**Number of people screened**	1964	2846	3431	2993	3187	2483	16,904
Number of people identified with TB symptoms	1590	2539	3126	2641	2817	2119	14,832
Number of people identified with TB symptoms tested for TB	861	1468	1933	1603	1597	1161	8623
Number of people with microbiologically confirmed TB (Bac+)	137	144	184	172	147	104	888
Number of people diagnosed with TB (all forms)	284	302	324	327	256	157	1650
Number of Bac+ TB patients started on treatment	135	142	180	162	143	100	862
Number of all forms TB patients started on treatment	279	296	316	311	250	148	1600
Number of Bac+ TB patients successfully treated	130	127	172	149	134	87	799
Number of all forms TB patients successfully treated	273	279	308	293	235	131	1519
							
% tested of those with TB symptoms	54%	58%	62%	61%	57%	55%	58%
Pre-treatment lost to follow-up (All Forms)	2%	2%	2%	5%	2%	6%	3%
Treatment Success Rate (All Forms)	98%	94%	97%	94%	94%	89%	95%

Notes: Bac+, microbiologically-confirmed cases.

**Table 2:** Notifications in the National TB Elimination Program in the public project intervention area between January 2016 and December 2018.

TB Case	Historical Baseline Notifications						Implementation Period Notifications						Additional Cases	Change from BASELINE (%)
	2016				2017		2017		2018					
	Q1	Q2	Q3	Q4	Q1	Q2	Q3	Q4	Q1	Q2	Q3	Q4		
All Forms	141	170	121	100	116	131	228	266	283	304	292	136	730	94%
Microbiologically-confirmed	66	66	59	44	55	50	74	97	98	142	110	86	267	79%

Note: The additional cases are the difference between the implementation period and the historical baseline notifications.

**Table 3:** Outcomes in the private project disaggregated by intervention between July 2017 and December 2018.

Indicators	Intervention		Total
	Diagnostic Hub	Private Provider	
**Number of people identified with TB symptoms**	12,041	0	12,041
Number of people identified with TB symptoms tested for TB	12,041	0	12,041
Number of people with microbiologically confirmed TB (Bac+)	2193	947	3140
Number of people diagnosed with TB (all forms)	2403	3362	5765
Number of Bac+ TB patients started on treatment	2193	947	3140
Number of all forms TB patients started on treatment	2403	3362	5765
Number of Bac+ TB patients successfully treated ^#^	891	168	1059
Number of all forms TB patients successfully treated ^#^	1791	716	2507
			
% tested of those with TB symptoms	100%	-	100%
Pre-treatment lost to follow-up (All Forms)	0%	0%	0%
Treatment Success Rate (All Forms) ^#^	75%	21%	43%

Notes: Bac+, microbiologically confirmed cases. ^#^ The treatment success figures are incomplete, as the project ended before the treatment outcomes could be reported for all of the patients who initiated treatment. After the project ended, the patients still on the treatment continued their treatment under the supervision of their health provider.

**Table 4:** Notifications in the National TB Elimination Program in the private project intervention area between January 2016 and December 2018.

	Historical Baseline Notifications						Implementation Period Notifications						Additional Cases	Change from BASELINE (%)
	2016				2017		2017		2018					
	Q1	Q2	Q3	Q4	Q1	Q2	Q3	Q4	Q1	Q2	Q3	Q4		
All forms (private)	464	490	674	657	821	825	869	1202	1446	2743	2142	1299	5770	147%
All forms (public)	2509	2503	2404	2486	2699	2425	2403	2533	2458	2776	2857	2877	878	6%

Note: The additional cases are the difference between the implementation period and historical baseline notifications.

## Data Availability

All of the data used for the analysis is available in the paper.

## References

[1] World Health Organization (2019). Global Tuberculosis Report 2019.

[2] Cazabon D., Alsdurf H., Satyanarayana S., Nathavitharana R., Subbaraman R., Daftary A., Pai M. (2017). Quality of Tuberculosis Care in High Burden Countries: The Urgent Need to Address Gaps in the Care Cascade. Int. J. Infect. Dis..

[3] Odone A., Roberts B., Dara M., van den Boom M., Kluge H., McKee M. (2018). People- and Patient-Centred Care for Tuberculosis: Models of Care for Tuberculosis. Int. J. Tuberc. Lung Dis..

[4] George M. (2020). The Fragmentation and Weakening of Institutions of Primary Healthcare. Econ. Polit. Wkly..

[5] Yellapa V., Devadasan N., Krumeich A., Pant Pai N., Vadnais C., Pai M., Engel N. (2017). How Patients Navigate the Diagnostic Ecosystem in a Fragmented Health System: A Qualitative Study from India. Glob. Health Action.

[6] Engel N., Ganesh G., Patil M., Yellappa V., Pant Pai N., Vadnais C., Pai M. (2015). Barriers to Point-of-Care Testing in India: Results from Qualitative Research across Different Settings, Users and Major Diseases. PLoS ONE.

[7] Storla D.G., Yimer S., Bjune G.A. (2008). A Systematic Review of Delay in the Diagnosis and Treatment of Tuberculosis. BMC Public Health.

[8] Bhatnagar H. (2019). User-Experience and Patient Satisfaction with Quality of Tuberculosis Care in India: A Mixed-Methods Literature Review. J. Clin. Tuberc. Mycobact. Dis..

[9] Mukerji R., Turan J.M. (2020). Challenges in Accessing and Utilising Health Services for Women Accessing DOTS TB Services in Kolkata, India. Glob. Public Health.

[10] Oxlade O., Murray M. (2012). Tuberculosis and Poverty: Why Are the Poor at Greater Risk in India?. PLoS ONE.

[11] Chandra A., Kumar R., Kant S., Parthasarathy R., Krishnan A. (2020). Direct and Indirect Patient Costs of Tuberculosis Care in India. Trop. Med. Int. Health.

[12] Yellappa V., Lefèvre P., Battaglioli T., Narayanan D., Van der Stuyft P. (2016). Coping with Tuberculosis and Directly Observed Treatment: A Qualitative Study among Patients from South India. BMC Health Serv. Res..

[13] Mason P.H., Roy A., Spillane J., Singh P. (2016). Social, Historical and Cultural Dimensions of Tuberculosis. J. Biosoc. Sci..

[14] Garg T., Gupta V., Sen D., Verma M., Brouwer M., Mishra R., Bhardwaj M. (2020). Prediagnostic Loss to Follow-up in an Active Case Finding Tuberculosis Programme: A Mixed-Methods Study from Rural Bihar, India. BMJ Open.

[15] Mukerji R., Turan J.M. (2018). Exploring Manifestations of TB-Related Stigma Experienced by Women in Kolkata, India. Ann. Glob. Health.

[16] Ali-Faisal S.F., Colella T.J.F., Medina-Jaudes N., Benz Scott L. (2017). The Effectiveness of Patient Navigation to Improve Healthcare Utilization Outcomes: A Meta-Analysis of Randomized Controlled Trials. Patient Educ. Couns..

[17] Peart A., Lewis V., Brown T., Russell G. (2018). Patient Navigators Facilitating Access to Primary Care: A Scoping Review. BMJ Open.

[18] Calhoun E.A., Esparza A. (2018). Patient Navigation: Overcoming Barriers to Care.

[19] Carter N., Valaitis R.K., Lam A., Feather J., Nicholl J., Cleghorn L. (2018). Navigation Delivery Models and Roles of Navigators in Primary Care: A Scoping Literature Review. BMC Health Serv. Res..

[20] Dalton M., Holzman E., Erwin E., Michelen S., Rositch A.F., Kumar S., Vanderpuye V., Yeates K., Liebermann E.J., Ginsburg O. (2019). Patient Navigation Services for Cancer Care in Low-and Middle-Income Countries: A Scoping Review. PLoS ONE.

[21] Bernardo B.M., Zhang X., Hery C.M.B., Meadows R.J., Paskett E.D. (2019). The Efficacy and Cost-Effectiveness of Patient Navigation Programs across the Cancer Continuum: A Systematic Review. Cancer.

[22] Sinha P., Shenoi S.V., Friedland G.H. (2019). Opportunities for Community Health Workers to Contribute to Global Efforts to End Tuberculosis. Glob. Public Health.

[23] Blok L., Creswell J., Stevens R., Brouwer M., Ramis O., Weil O., Klatser P., Sahu S., Bakker M.I. (2014). A Pragmatic Approach to Measuring, Monitoring and Evaluating Interventions for Improved Tuberculosis Case Detection. Int. Health.

[24] (2011). Registrar General of India Census of India 2011.

[25] (2011). Socio-Economic Caste Census 2011.

[26] (2011). Annual Health Survey 2011-12, Bihar Factsheet.

[27] International Institute for Population Sciences NFHS 4 Factsheet Samastipur. http://rchiips.org/nfhs/factsheet_nfhs-4.shtml.

[28] Central TB Division (2018). India TB Report 2018: Revised National Tuberculosis Control Program—Annual Status Report.

[29] Central TB Division (2019). India TB Report 2019: Revised National Tuberculosis Control Program—Annual Status Report.

[30] Garg T., Bhardwaj M., Deo S. (2020). Role of Community Health Workers in Improving Cost Efficiency in an Active Case Finding Tuberculosis Programme: An Operational Research Study from Rural Bihar, India. BMJ Open.

[31] Office of the Registrar General, India (2020). SRS Bulletin, Sample Registration System.

[32] Office of the Registrar General, India (2019). Special Bulletin on Maternal Mortality in India 2015-17, Sample Registration System.

